# Air pollution exposure and cataract outcomes: a systematic review and pollutant-specific meta-analysis

**DOI:** 10.3389/fmed.2026.1969759

**Published:** 2026-10-01

**Authors:** Bin Zhang, Bing Xu, Kasra Pakdaman

**Affiliations:** 1 Department of Cataract, Lanzhou Aier Eye Hospital, Lanzhou, China; 2 Department of Glaucoma, Lanzhou Aier Eye Hospital, Lanzhou, China; 3 School of Nursing and Midwifery, Mazandaran University of Medical Sciences, Sari, Iran

**Keywords:** environmental ophthalmology, environmental risk factors, lens opacity, ocular epidemiology, traffic emissions

## Abstract

**Background:**

Air pollution is increasingly recognized as a potentially modifiable environmental determinant of age-related ocular disease, but its association with cataract remains incompletely characterized. Epidemiological evidence remains heterogeneous across pollutants and study methods, although emerging experimental findings support biological plausibility for pollution-related lens injury.

**Objectives:**

To systematically evaluate the epidemiologic evidence linking air-pollution exposure with cataract-related outcomes and to quantitatively synthesize pollutant-specific associations where sufficiently comparable data were available.

**Methods:**

PubMed, Embase, Scopus, Web of Science, and Google Scholar were searched through August 10, 2026 for observational studies reporting cataract-specific associations with specific air pollutants. Risk of bias was assessed using ROBINS-E for longitudinal follow-up studies and the WHO air-quality epidemiology instrument for cross-sectional and aggregate studies. Comparable hazard ratios (HRs) were synthesized using restricted maximum-likelihood random-effects models in Stata 18, with Knapp–Hartung sensitivity analyses.

**Results:**

Fifteen studies were included in the systematic review, of which seven contributed to at least one meta-analysis. The pooled HR was 1.036 (95% CI, 0.988–1.086) per 1 µg/m^3^ increase in PM₂.₅, 1.041 (95% CI, 1.013–1.070) per 10 µg/m^3^ increase in NO₂, and 1.010 (95% CI, 0.983–1.038) per study-specific interquartile-range increase in black carbon. Heterogeneity was substantial for PM₂.₅ (I^2^ = 99.68%) and NO₂ (I^2^ = 86.04%) but lower for black carbon (I^2^ = 33.41%). The highest-versus-lowest quartile analysis also showed a positive association for NO₂ (HR, 1.094; 95% CI, 1.057–1.133; I^2^ = 0.11%), whereas corresponding PM₂.₅ and PM₁₀ estimates were not statistically significant. Knapp–Hartung adjustment widened the NO₂ confidence intervals, indicating sensitivity to the small number of contributing studies.

**Conclusion:**

NO₂ showed the most consistent association with cataract outcomes, whereas evidence for PM₂.₅ remained heterogeneous and that for black carbon was limited. Although these findings support further investigation of traffic-related and other ambient pollution mixtures in cataract epidemiology, the sensitivity of NO₂ estimates to small-study inference precludes firm causal conclusions. Independent prospective studies with improved exposure assessment and standardized cataract phenotyping are needed.

**Systematic Review Registration:**

https://www.crd.york.ac.uk/PROSPERO/recorddashboard#, identifier CRD420261509514.

## 1 Introduction

Cataract remains a major global cause of blindness and vision impairment and an increasingly important consequence of population ageing. In 2020, cataract accounted for an estimated 17.0 million people with blindness and 83.5 million with moderate-to-severe vision impairment worldwide, while the absolute number affected continued to rise despite reductions in age-standardized cataract blindness (1). Ambient air pollution represents a major global public-health burden (2). Biological plausibility for ocular effects is also emerging, as experimental and human lens data indicate that PM₂.₅ exposure can disrupt redox homeostasis, promote lipid peroxidation, and activate ferroptosis-related pathways in lens tissue (3). These converging burdens have increased interest in air pollution as a potentially modifiable environmental determinant of cataract.

Epidemiologic evidence linking air pollution to cataract remains limited. Previous evidence syntheses have consistently characterized the cataract literature as sparse and heterogeneous, with insufficient data for robust pollutant-specific synthesis, while a more recent meta-analysis also found the PM₂.₅–cataract association inconclusive (4–6). These limitations support the need for an updated, cataract-focused quantitative synthesis across individual pollutants.

The evidence base has expanded materially in recent years. Two national Chinese cohorts reported prospective associations between long-term PM₂.₅ exposure and age-related cataract (7), while a nationwide Danish cohort of more than three million adults identified senile cataract among outcomes associated with long-term PM₂.₅ and nitrogen dioxide (NO₂) exposure (8). More recent United Kingdom (UK) Biobank research has evaluated joint exposure to multiple pollutants in relation to incident cataract (9). Prospective French cohorts have further extended the literature to PM₂.₅, NO₂, black carbon, and incident cataract surgery (10). These studies broaden the geographic, exposure, and clinical scope of the literature and warrant a synthesis centered specifically on cataract.

A cataract-focused, pollutant-specific synthesis is needed because chemically distinct pollutants should not be assumed to have equivalent ocular effects, and because cataract diagnosis and cataract surgery represent related but clinically non-equivalent outcomes. Separating pollutant-specific evidence also avoids obscuring potentially different associations within a single composite “air pollution” effect. Accordingly, this systematic review and meta-analysis aimed to synthesize the epidemiologic evidence relating exposure to specific air pollutants to cataract outcomes and to quantify pollutant-specific associations where the evidence permitted. A further objective was to define the consistency and remaining uncertainty of this evidence base and thereby identify priorities for future environmental ophthalmic research.

## 2 Materials and methods

### 2.1 Study design

This systematic review and meta-analysis was conducted and reported in accordance with the Preferred Reporting Items for Systematic Reviews and Meta-Analyses (PRISMA) 2020 statement (11). This study was registered in PROSPERO with ID of [CRD420261509514].

### 2.2 Eligibility criteria

Studies were eligible if they evaluated the association between exposure to specific air pollutants and a cataract-specific outcome in human populations. Eligible exposures included particulate matter (PM₂.₅, PM₁₀, PM₂.₅–₁₀, and related particulate metrics), nitrogen dioxide (NO₂), nitrogen oxides (NOx), nitric oxide (NO), ozone (O₃), sulfur dioxide (SO₂), carbon monoxide (CO), and black carbon or related pollutant-specific proxies. Exposure could be assessed using monitoring data, modeled or satellite-derived concentrations, land-use regression, or other pollutant-specific exposure metrics. Studies in which exposure was defined solely by household cooking-fuel type, biomass or solid-fuel use, or non-specific indoor cooking smoke without a pollutant-specific exposure estimate were not considered eligible.

Studies were excluded if they did not report a cataract-specific outcome; combined cataract with other ocular diseases without separately extractable cataract data; reported only disease-burden measures such as disability-adjusted life-years without cataract occurrence, prevalence, incidence, or surgery data; or represented duplicate or overlapping reports without additional eligible information. Eligibility for the quantitative meta-analysis was assessed separately from eligibility for the systematic review. Meta-analysis was restricted to sufficiently comparable adjusted hazard ratios (HRs) addressing the same pollutant and exposure contrast. Other quantitative measures, including odds ratios, Pearson correlation coefficients, multinomial-logit coefficients, and fuzzy-cognitive-map weights, were retained for the systematic review but were not combined with HRs in the primary meta-analyses.

### 2.3 Information sources and search strategy

PubMed, Embase, Scopus, Web of Science, and Google Scholar were searched through August 10, 2026. The search combined controlled vocabulary and free-text terms related to air pollution and specific pollutants with terms for cataract, lens opacity, and cataract surgery. Reference and citation searching was additionally used to identify potentially eligible reports. The database-specific search strategies and numbers of retrieved records are provided in Supplementary Table S1. Accordingly, the search strategy was intentionally pollutant-focused and did not use biomass-, solid-fuel-, or cooking-fuel-specific terms, because source-based fuel-use proxies alone were outside the predefined exposure criteria (12).

### 2.4 Study selection and data extraction

Retrieved records were screened first by title and abstract and subsequently by full-text assessment against the predefined eligibility criteria. Reasons for full-text exclusion were recorded and summarized in the PRISMA flow diagram. Two reviewers (K.P. and B.Z.) independently screened titles and abstracts and subsequently assessed potentially eligible full texts. Disagreements were resolved through discussion and, when necessary, consultation with a third reviewer (B.X.).

For each included study, data were extracted into a structured database, including first author, publication year, country, study design and period, source population, sample size, number of cataract cases or events, age, follow-up duration, cataract definition and ascertainment method, air-pollution exposure and assessment method, exposure window and unit, effect measure, 95% confidence interval (CI), and covariates included in the statistical model. Data extraction was performed independently by K.P. and B.Z., with discrepancies resolved by consensus.

For quantitative synthesis, one independent estimate was selected from each study or underlying cohort for each pooled estimand. When multiple estimates were available, we preferentially selected the most fully adjusted estimate for the overall study population and the outcome and exposure window most closely aligned with the prespecified pollutant–outcome estimand, irrespective of statistical significance. Continuous, quartile-based, and threshold-based estimates were analyzed separately. Alternative exposure windows, sensitivity models, subtype estimates, and other correlated estimates were retained in the extraction dataset but were not treated as independent observations within the same meta-analysis.

Potential overlap between reports derived from the same underlying cohorts was assessed before pooling. In particular, multiple reports based on the UK Biobank and reports containing participants from the Chinese Longitudinal Healthy Longevity Survey were not treated as independent when they contributed to the same pooled estimand. A single compatible estimate was selected from an overlapping source population for each pollutant-specific meta-analysis.

### 2.5 Risk-of-bias assessment

Risk of bias was assessed according to study design. Longitudinal non-randomized follow-up studies of exposure effects were evaluated using the 2024 Risk Of Bias In Non-randomized Studies of Exposures (ROBINS-E) framework (13). ROBINS-E evaluates bias arising from confounding, exposure measurement, selection of participants into the study or analysis, post-exposure interventions, missing data, outcome measurement, and selection of the reported result. Cross-sectional and aggregate/ecological air-pollution studies were evaluated using the World Health Organization risk-of-bias instrument developed for epidemiological studies informing the WHO Global Air Quality Guidelines (14). This assessment considered confounding, selection bias, exposure assessment, outcome measurement, missing data, and selective reporting. Risk-of-bias judgments were reported by domain rather than converted into a numerical quality score. The detailed assessments are presented in Supplementary Table S2.

### 2.6 Statistical analysis

All statistical analyses were performed using Stata version 18.0 (StataCorp LLC, College Station, TX, USA). Effect estimates eligible for quantitative synthesis were analyzed as HRs on the logarithmic scale using inverse-variance weighting, with standard errors derived from reported 95% confidence intervals when not directly available (15). To improve comparability across studies, continuous estimates were harmonized to common exposure increments before pooling, assuming a log-linear association over the relevant exposure range; PM₂.₅ estimates were expressed per 1 µg/m^3^ increase and NO₂ estimates per 10 µg/m^3^ increase, whereas black-carbon estimates were retained per study-specific interquartile-range increase because exposure metrics were not directly interchangeable across studies. Categorical contrasts, including Q4 versus Q1 and study-specific NO₂ thresholds, were analyzed separately without conversion to continuous increments. Random-effects models with restricted maximum-likelihood (REML) estimation were used to account for between-study heterogeneity (16), which was assessed using Cochran's Q and quantified with the I^2^ statistic (17). Because incident cataract diagnosis and cataract surgery represent related but clinically non-equivalent endpoints, pooled models were interpreted as summary associations with cataract-related events rather than as effects on a single uniform clinical endpoint. Studies were combined only when the pollutant, exposure contrast, effect measure, and longitudinal analytic framework were otherwise sufficiently comparable. Outcome definition was examined as a potential source of heterogeneity where the number of available studies permitted; PM₂.₅ was the only continuous-exposure analysis with at least two studies in both diagnosis- and surgery-based outcome strata. An outcome-based subgroup analysis was performed for PM₂.₅, comparing incident cataract diagnosis with incident cataract surgery, and subgroup differences were formally tested. Leave-one-out analyses were conducted for the primary PM₂.₅, NO₂, and black-carbon models. Because few studies contributed to each pooled analysis, Knapp–Hartung-adjusted inference was additionally examined as a sensitivity analysis (18). Formal assessment of funnel-plot asymmetry or publication bias was not undertaken because all pollutant-specific meta-analyses included fewer than 10 studies, for which such tests have limited power and may yield unreliable inferences (19). All statistical tests were two-sided, with *P* < 0.05 considered statistically significant.

## 3 Results

### 3.1 Study selection

The database search identified 1,066 records, with four additional records identified through citation searching. After removal of 288 duplicates, 778 records underwent title and abstract screening, of which 748 were excluded. Thirty reports were sought for full-text retrieval; two could not be retrieved and 28 were assessed for eligibility. Thirteen reports were excluded after full-text assessment. All four reports identified through citation searching were retrieved and assessed but were subsequently excluded. Overall, 15 studies were included in the systematic review, of which seven contributed to at least one quantitative meta-analysis (Figure 1).

**Figure 1:**
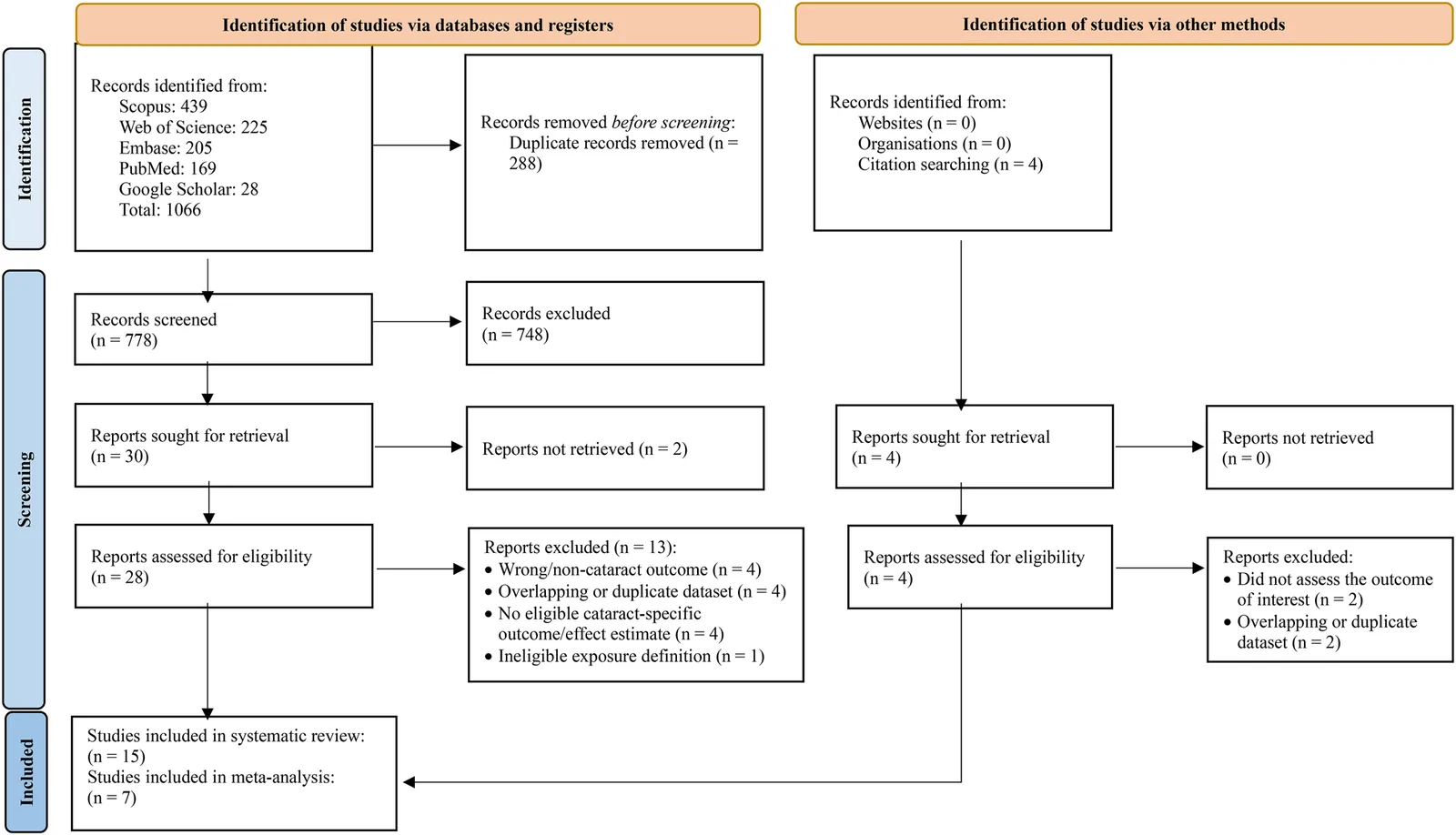
PRISMA 2020 flow diagram of study selection. Adapted from the PRISMA 2020 flow diagram template (11), licensed under CC BY 4.0.

### 3.2 Study characteristics

The 15 included studies were published between 2018 and 2026 and were conducted in South Korea, Canada, the United Kingdom, China, Spain, Denmark, and France (Table 1). Eleven studies used longitudinal or follow-up designs, two were cross-sectional studies, and two used ecological or aggregate designs. Study populations ranged from 829 participants in the 3City-Alienor cohort to more than 3 million individuals in the nationwide Danish cohort. Cataract ascertainment varied across studies and included slit-lamp examination with standardized lens grading, self-reported physician diagnosis, hospital or registry-based diagnostic codes, incident cataract surgery, and aggregate cataract-surgery records. Exposure assessment included residential land-use regression, ambient monitoring networks, satellite- and model-based estimates, and regional or provincial exposure assignment. The pollutants most frequently evaluated included PM₂.₅, PM₁₀, NO₂/NOx, O₃, SO₂, CO, and black carbon or PM₂.₅ absorbance. Seven studies contributed comparable effect estimates to the quantitative syntheses; studies with non-comparable outcome definitions, exposure contrasts, effect measures, or overlapping analytic populations were retained in the systematic review but were not combined within the same pooled estimand.

**Table 1:** Characteristics of studies included in the systematic review (*n* = 15).

Study	Country	Design/study period	Data source/population	Study/analysis N (cataract cases/events)	Age	Follow-up	Cataract outcome ascertainment	Exposure assessment	Air-pollution exposure(s)	Effect measure	Main covariate adjustment
Choi et al. (2018) (26)	South Korea	Population-based cross-sectional; 2008–2012	KNHANES IV–V; Adults >40 years	19,910 (10,085)	58.9 ± 12.0 years	Not applicable	Ophthalmologist slit-lamp examination; LOCS III; prior cataract surgery counted as cataract-positive	Ambient monitoring data from Korea Ministry of Environment; 283 monitoring stations; exposure assigned at 16 administrative-division level	NO2, O3, PM10, SO2	OR	Age, sex, residence, education, income, sun exposure, smoking, alcohol, hypertension, diabetes, hypercholesterolemia, myopia, obesity
Shin et al. (2020)^a^ (24)	South Korea	Nationwide population-based retrospective cohort study; Baseline/washout 2002–2003; incident outcome follow-up 1 Jan 2004–31 Dec 2015	Korean National Health Insurance Service–National Sample Cohort (NHIS–NSC); Adults aged ≥50 years in 2002, cataract-free during 2002–2003, with stable/matched residence and smoking data	115,728 (16,814)	Mean 60.0 ± 7.2 years	Up to 12 years (2004–2015)	New cataract required both physician diagnosis (ICD-10 H25/H26) and cataract surgery code S5119; participants with cataract diagnosis in 2002–2003 were excluded. Index date was first cataract diagnosis.	Residential-area exposure matched to 268 nationwide surveillance stations using five-digit residence codes. Hourly monitoring data were summarized as average concentrations and categorized into quartiles. PM10/NO2/CO/SO2/O3 were available from 2002 to 2015; PM2.5 was available only for 2015.	CO, NO2, O3, PM10, PM2.5, SO2	HR	Age, sex, smoking status, income/insurance level, urbanization, and comorbidity
Chua et al. (2021)^a^ (23)	United Kingdom	Prospective observational cohort; Baseline 2006–2010; follow-up to 31 Mar 2017	UK Biobank participants aged 40–69 at enrollment; baseline cataract and early/pre-2011 surgery excluded	433,727 (16,307)	Not reported overall; cases 62.5 ± 5.6, controls 56.0 ± 8.1 years	Mean 94 ± 15 months (7.8 years)	Incident first-eye cataract surgery via NHS linkage; OPCS-4 C71.2 (phacoemulsification) or C75.1 (lens replacement). Excluded baseline cataract, surgery within 1 year of baseline, and surgery in 2010 or earlier.	ESCAPE land-use regression at baseline residential address using GIS predictors. NO2 averaged 2005/2006/2007/2010; PM10 averaged 2007/2010; PM2.5, PM2.5-10, PM2.5 absorbance and NOx based on 2010 estimates.	NO2, NOx, PM10, PM2.5, PM2.5 absorbance (black carbon proxy), PM2.5–10	HR	Age, sex, race, Townsend deprivation index, BMI, smoking status, diabetes
Grant et al. (2021) (20)	Canada	Cross-sectional analysis of baseline cohort data; Baseline assessments Dec 2011–Jul 2015	Canadian Longitudinal Study on Aging (CLSA) Comprehensive Cohort; Community-dwelling adults aged 45–85 years near 11 CLSA data collection sites in 7 Canadian provinces	29,147 (8,286)^b^	Not reported	Not applicable	Self-reported prior physician diagnosis of cataract at baseline	Annual mean ambient pollutants assigned to participant postal code through CANUE. PM2.5 was satellite-derived (AOD + GEOS-Chem) calibrated to ground observations; O3 from Environment and Climate Change Canada air-quality modelling; SO2 from OMI satellite/GEM profiles; NO2 from national monitoring plus land-use regression/local gradients.	O3, PM2.5	OR	Age, sex, ethnicity, education, household income, smoking, diabetes, hypertension, province
Wu et al. (2022)^a^ (21)	United Kingdom	Prospective cohort study; UKB recruitment 2006–2010; follow-up through 30 Nov 2020	UK Biobank participants free of age-related cataract at baseline; exclusions also included congenital lens malformations, vision loss, eye loss, prior eye surgery, and missing exposure/covariate data	291,627 (12,826)	Mean 55.83 ± 8.1 years	Mean 11.3 ± 1.8 years	Incident hospital inpatient diagnosis of age-related cataract after enrolment: ICD-10 H25. Nuclear cataract: H25.1. Non-nuclear cataract: H25.0, H25.2, H25.8, H25.9. Follow-up ended at first cataract event, death, loss to follow-up, or 30 Nov 2020.	Annual mean residential PM2.5 concentration for 2010 estimated with ESCAPE land-use regression using GIS-derived traffic and land-use variables; participant baseline residential address was matched to the exposure surface. NO2 from ESCAPE LUR was included as a covariate.	PM2.5	HR	Age, sex, ethnicity, socioeconomic and lifestyle factors, BMI, ocular/systemic comorbidities, cataract polygenic risk score, NO₂, and physical activity.
Li et al. (2023)^a^ (7)	China	Longitudinal population-based cohort analysis of two national cohorts; CFPS baseline survey with follow-ups in 2012, 2014, 2016, 2018; CLHLS 1998–2018	China Family Panel Studies (CFPS) + Chinese Longitudinal Healthy Longevity Survey (CLHLS); Adults >50 years, cataract-free at baseline, with ≥1 follow-up; main analysis additionally required ≥3 years follow-up and complete key information	22,298 (1,897)	Mean not reported; 36.3% age 50–65 and 63.7% > 65 years	≥3 years required for main analysis; follow-up through 2018	Incident age-related cataract based on self-report of a physician diagnosis at follow-up: “Have you been diagnosed with cataracts by your doctor?” Disease status was reassessed at each follow-up.	Annual ambient PM2.5 assigned by baseline city address using a spatiotemporal land-use regression model (population density, road length, land-use variables and ambient visibility); annual predictions were used to construct long-term exposure windows.	PM2.5	HR	Age, sex, BMI, annual household income, education, smoking, drinking, marital status, ethnicity
Echevarría-Lucas et al. (2024) (35)	Spain	Ecological longitudinal/spatiotemporal observational study; Cataracts 2011–2021; pollution 2010–2020	Andalusian Health Service (Malaga–Axarquía) + INE + REDIAM; Residents of 23 municipalities; analyses for ages 15–59 and ≥60 years	134,223 (9,487)	Not reported	11-year ecological series; temporal analyses use 1-year lag	Andalusian Health Service surgical records; operable cataract (visual acuity <0.4) followed by cataract surgery; standardized incidence calculated.	REDIAM county/municipality-level annual emissions; ecological exposure assignment. Pollution series 2010–2020 linked to cataract outcomes 2011–2021.	CH4, CO, CO2, N2O, PM10, PM2.5, Total pollutants	Pearson r	No covariate adjustment in bivariate pollution–cataract correlations; study additionally examined climate, occupational and socioeconomic variables.
Hegelund et al. (2024)^a^ (8)	Denmark	Prospective nationwide phenome-wide association cohort study; 1 Jan 2000–31 Dec 2017	Nationwide Danish population/ELAPSE-linked register cohort; All Danish residents aged ≥30 years on 1 Jan 2000 who had lived in Denmark ≥1 year	3,050,532 ^c^ (364,591)	Median 51.4 years (range 30.0–110.7)	Up to 18 years; H25 average follow-up 14.7 years	First registered diagnosis from the Danish National Patient Register and/or underlying cause of death; ICD-10 three-character outcomes. Cataract phenotypes extracted here: H25 senile cataract and H26 other cataract.	European-wide hybrid land-use regression at 100 × 100 m resolution using geocoded baseline residence; 2010 PM2.5 and NO2 surfaces were extrapolated back in time to represent long-term exposure at baseline.	NO2, PM2.5	HR	Age as underlying time axis; country of origin, cohabitation status, education, occupational status, household-size-equivalised disposable income; models stratified by sex and residential municipality.
Gayraud et al. (2025)^a^ (22)	France	Prospective population-based cohort; Baseline 1999–2000; follow-up through 2017	3C Bordeaux/Alienor (3City-Alienor); Bordeaux residents aged ≥65 years at 3C recruitment; no cataract surgery at baseline	829 (507)	72.57 ± 4.19 years	Median 14.1 years; range 6.5–17.9 years	First cataract surgery during follow-up; self-reported year of surgery and surgery status checked by slit-lamp at repeated eye examinations.	100 × 100 m ELAPSE land-use regression at geocoded residential address; 2010 models temporally extrapolated to 1990–2016; 10-year pre-baseline average assigned.	BC, NO2, PM2.5	HR	Age, sex, deprivation index, diabetes, asthma, hypertension, lifetime ambient UVR exposure, BMI, smoking status.
Li et al. (2025) (9)	United Kingdom	Prospective population-based cohort study; Recruitment 2006–2010; follow-up to 1 Sep 2023	UK Biobank adults recruited from 22 centers in England, Scotland and Wales; participants with cataract, glaucoma or AMD at baseline and those lacking air-pollution exposure were excluded	441,567 (55,104)	Mean 56.32 ± 8.09 years	Median 14.41 years (IQR 13.64–15.07)	Incident cataract identified during follow-up through linked self-report/clinical diagnosis, primary care, inpatient hospital and death records; ICD-10 H25 (senile cataract), H26 (other cataract) and H28 (cataract/lens disorders in diseases classified elsewhere).	2010 annual mean residential air-pollution estimates from ESCAPE land-use regression models using GIS predictors (traffic intensity, population, topography, land use), assigned to home address as proxies of long-term exposure. NO was derived as NOx minus NO2.	Air pollution score, NO, NO2, PM10, PM2.5, PM ₂.₅–₁₀	HR	Age, sex, ethnicity, assessment center, Townsend Deprivation Index, education, BMI, smoking status, alcohol intake frequency, hypertension, diabetes mellitus, hyperlipidemia
Miao et al. (2025) (36)	China	Aggregate hospital time-series/ecological modeling study using Fuzzy Cognitive Maps (preprint); 1 Jan 2019–22 Jul 2024	The Central Hospital of Wuhan cataract surgery records; Patients undergoing phacoemulsification with intraocular lens implantation at The Central Hospital of Wuhan	14,086 (14,086)	Mean 70.25 ± 9.4 years	No individual longitudinal follow-up; daily hospital surgery-volume time series	Daily/aggregate volume of cataract surgeries among hospital patients undergoing phacoemulsification with intraocular lens implantation; the paper frames the outcome as senile cataract surgery volume/timing rather than incident cataract risk in individuals.	City-level Wuhan weather and air-quality data from rp5.ru and aqistudy.cn over the corresponding period. Daily environmental values were derived from >10 measurements/day when available and min-max normalized before FCM modeling.	CO, NO2, O3, PM10, PM2.5, SO2	FCM mean simple-path weight	FCM jointly modeled multiple environmental variables (air pollutants plus temperature, pressure, humidity, wind, visibility, dew point and precipitation); no individual-level clinical or sociodemographic confounder adjustment.
Wang et al. (2025) (37)	United Kingdom	Prospective cohort study; UKB recruitment 2006–2010; analysis baseline set to 1 Jan 2010; median follow-up 12.4 years	UK Biobank — participants with baseline refractometry; UK Biobank adults with refractometry at baseline; participants lost to follow-up, with pre-existing cataract/glaucoma/diabetic retinopathy/retinal detachment/macular degeneration, or missing air-pollution exposure were excluded	114,930 (13,939)	Mean 56.9 ± 7.98 years	Median 12.4 years (reported spread 1.93 years)	Incident cataract identified from linked hospital admission/diagnosis records (England, Wales, Scotland) using ICD-9/ICD-10 codes; exact cataract codes are listed in Supplementary Table 1, which was not included in the supplied main PDF.	Annual 2010 residential concentrations estimated by ESCAPE land-use regression using GIS predictors including traffic intensity, land use and topography. PM2.5/PM10 values were unavailable for addresses >400 km from Greater London and those participants were excluded.	NO2, NOx, PM10, PM2.5	HR	Age, sex, ethnicity, education, BMI, smoking, alcohol intake, hypertension, and diabetes; TDI additionally included in threshold models.
Yuan et al. (2025) (25)	United Kingdom	Prospective population-based cohort with multi-state transition analysis; UKB recruitment 2006–2010; follow-up through 30 Sep 2023	UK Biobank adults free of cataract and dementia at baseline; exclusions for baseline cataract/dementia, missing air-pollution information, missing covariates, or cataract and dementia diagnosed on the same date	409,608 (51,786)	Mean 56.4 ± 8.1 years	Median 14.5 years (IQR 13.8–15.1)	Incident cataract ascertained using UK Biobank first-occurrence fields for ICD-10 H25, H26 and H28, linking self-reported medical conditions, hospital inpatient data and death-register records. Primary cataract transition was baseline to first incident cataract.	Annual 2010 residential concentrations estimated with ESCAPE land-use regression on 100 × 100 m grids using GIS predictors (population density, traffic intensity and land use), assigned to participants’ geocoded residential coordinates. 2010 annual averages were used as proxies for long-term exposure.	Air pollution score, NO2, NOx, PM10, PM2.5, PM2.5–10	HR	Age, sex, ethnicity, Townsend deprivation index, BMI, smoking, drinking, education, physical activity, diet, hypertension, diabetes and coronary heart disease.
Zhou et al. (2025) (27)	China	Longitudinal population-based cohort analysis (retrospective analysis of CLHLS cohort data); CLHLS surveys 1998–2018	Chinese Longitudinal Healthy Longevity Survey (CLHLS); CLHLS participants across 23 Chinese provincial-level regions with repeated follow-up; final analysis required at least 3 follow-up visits and complete key covariate data	11,449 (1,714)	Mean 81.07 ± 11.76 years; 94.31% aged ≥65 years	Repeated CLHLS follow-up from enrollment to cataract self-report, death, withdrawal, or end of follow-up through 2018	Age-related cataract based on repeated self-report of a doctor diagnosis. At baseline and follow-up surveys, participants were asked whether they had received a doctor diagnosis of cataract; follow-up responses were used to track onset.	Annual maximum daily 8-hour mean ozone (MDA8 O3) derived from GBD 2019 using TOAR and CNEMC data, chemical-transport modeling with Bayesian maximum entropy, and NASA G5NR-Chem downscaling. Because individual addresses were unavailable, participants were assigned provincial-average MDA8 O3 as a surrogate exposure.	O3 (MDA8)	HR	age, sex, BMI, annual household income, education, smoking status, drinking status, marital status, ethnicity, and type 2 diabetes.
Gayraud et al. (2026)^a^ (10)	France	Pooled analysis of two prospective cohorts; Gazel 2007–2017; Constances 2012–2021	Constances + Gazel (pooled within report); Two nationwide French prospective cohorts; participants age >53 years; baseline cataract surgery excluded	36,140 (5,543)	60.16 ± 4.59 years	10 years	Incident cataract surgery from French SNDS procedure records; first surgery date used (phacoemulsification procedure codes).	ELAPSE 100 × 100 m land-use regression at geocoded residential histories; 2010 surfaces annually rescaled; individual 10-year pre-baseline average exposure.	BC, NO2, PM2.5	HR	Age, sex, education level, and study cohort (Constances vs. Gazel)

BC, black carbon; CLHLS, Chinese Longitudinal Healthy Longevity Survey; FCM, fuzzy cognitive map; HR, hazard ratio; LUR, land-use regression; MDA8, maximum daily 8-hour mean; NO, nitric oxide; NO₂, nitrogen dioxide; NOx, nitrogen oxides; O₃, ozone; OR, odds ratio; PM₂.₅, particulate matter ≤2.5 μm; PM₂.₅–₁₀, coarse particulate matter 2.5–10 μm; PM₁₀, particulate matter ≤10 μm; SO₂, sulfur dioxide.

a Studies contributing to at least one quantitative meta-analysis (*n* = 7) are marked with an alphabet (^a^).

b 28,550 participants had non-missing cataract status; regression N varied according to covariate completeness.

c Maximum cohort size varied by phenotype; N shown corresponds to the H25 senile-cataract analysis.

### 3.3 Risk of bias

Among the 11 longitudinal studies assessed using ROBINS-E, two were judged to have low overall risk of bias, eight had some concerns, and one was rated as high risk. The principal domains contributing to concerns were confounding, exposure assessment, participant selection, and selective reporting. Among the four cross-sectional or aggregate studies assessed using the WHO air-quality epidemiology risk-of-bias instrument, two were rated as having moderate overall risk of bias and two as high risk (Supplementary Table S2).

### 3.4 Continuous-exposure meta-analyses

Five studies contributed to the primary PM₂.₅ meta-analysis after effect estimates were harmonized to a 1-µg/m^3^ increase in PM₂.₅. The random-effects pooled HR was 1.036 (95% CI, 0.988–1.086; *P* = 0.15), with substantial between-study heterogeneity (I^2^ = 99.68%) (Figure 2). Three studies contributed continuous NO₂ estimates standardized to a 10-µg/m^3^ increase. The pooled HR was 1.041 (95% CI, 1.013–1.070), with I^2^ = 86.04%. For black carbon, three studies contributed estimates expressed per study-specific interquartile range (IQR) increase, yielding a pooled HR of 1.010 (95% CI, 0.983–1.038; *P* = 0.47; I^2^ = 33.41%) (Figure 2).

**Figure 2:**
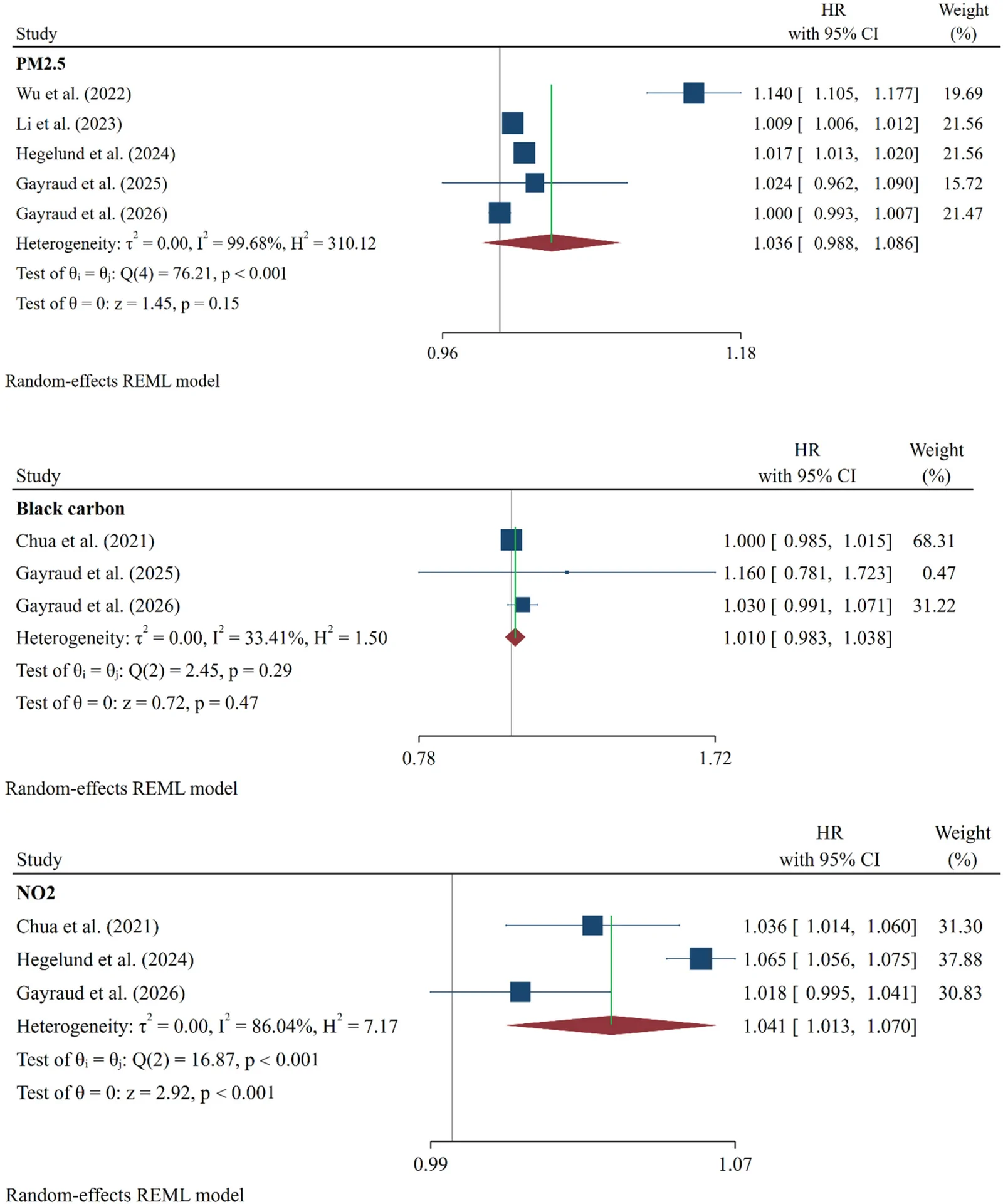
Random-effects meta-analyses of continuous associations between ambient air-pollutant exposure and cataract outcomes. PM₂.₅: HR per 1 µg/m^3^ increase, NO₂: HR per 10 µg/m^3^ increase, Black carbon: HR per study-specific IQR increase. An HR >1 indicates a higher rate of the cataract-related outcome with greater pollutant exposure, whereas an HR <1 indicates a lower rate.

### 3.5 Highest-versus-lowest exposure analyses

Two studies provided comparable highest-versus-lowest quartile estimates for each of PM₂.₅, PM₁₀, and NO₂ (Figure 3). For PM₂.₅, the pooled HR comparing Q4 with Q1 was 1.029 (95% CI, 0.822–1.288; *P* = 0.80; I^2^ = 86.43%). The corresponding estimate for PM₁₀ was 1.041 (95% CI, 0.984–1.100; *P* = 0.16; I^2^ = 65.87%). For NO₂, the pooled Q4-versus-Q1 HR was 1.094 (95% CI, 1.057–1.133), with minimal heterogeneity (I^2^ = 0.11%). A separate analysis of two studies using study-specific NO₂ thresholds yielded a pooled HR of 1.214 (95% CI, 0.881–1.673; *P* = 0.24), with I^2^ = 86.34% (Supplementary Figure S1).

**Figure 3:**
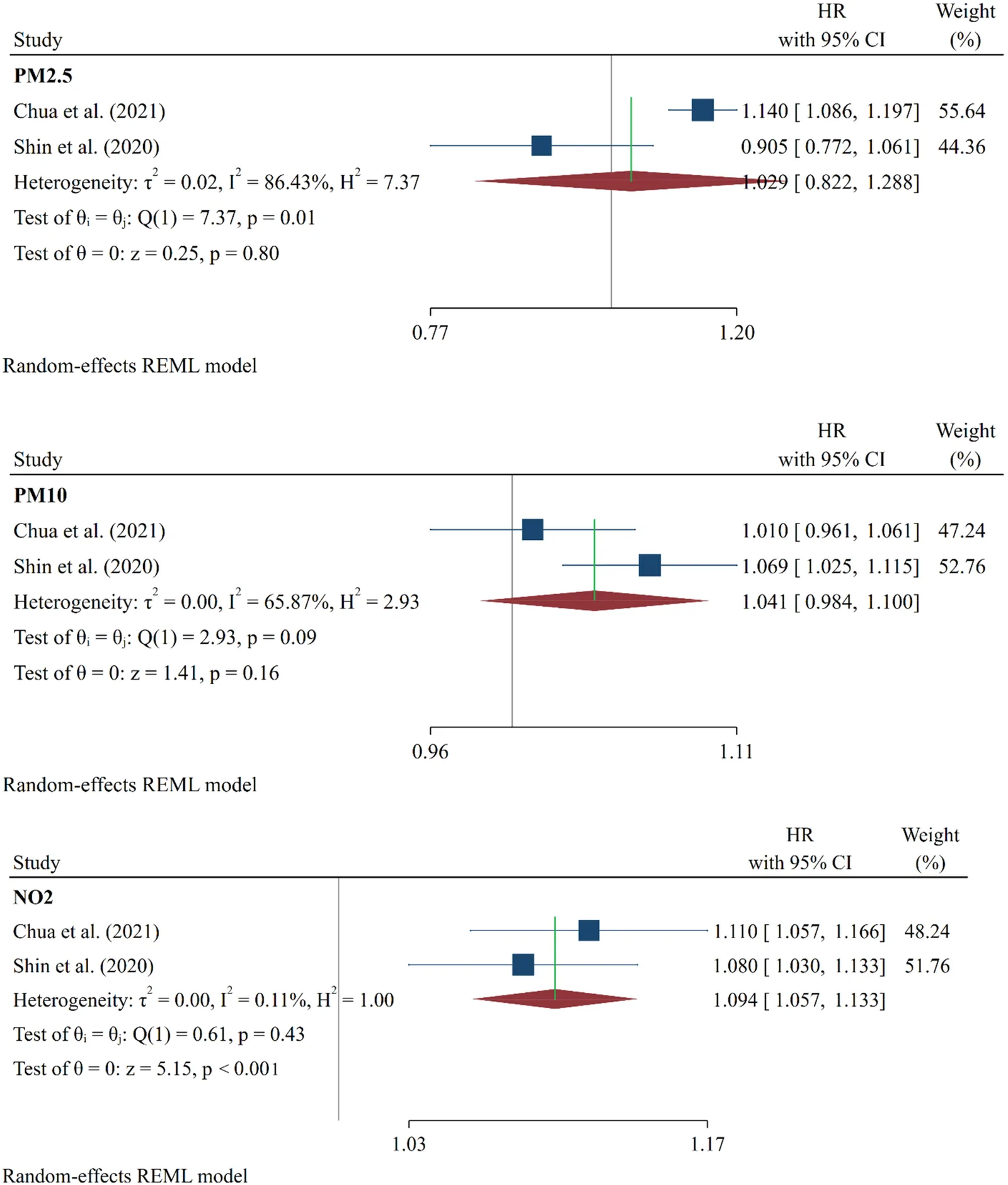
Random-effects meta-analyses comparing the highest versus lowest quartiles of ambient air-pollutant exposure and cataract outcomes. PM₂.₅: HR for Q4 vs. Q1, PM₁₀: HR for Q4 vs. Q1, NO₂: HR for Q4 vs. Q1. An HR >1 indicates a higher rate of the cataract-related outcome with greater pollutant exposure, whereas an HR <1 indicates a lower rate.

### 3.6 Subgroup and sensitivity analyses

In the PM₂.₅ subgroup analysis according to outcome definition, the pooled HR was 1.052 (95% CI, 0.975–1.135; I^2^ = 99.87%) for incident cataract diagnosis and 1.000 (95% CI, 0.993–1.008; I^2^ = 0.14%) for incident cataract surgery. The test for subgroup differences was not statistically significant (*P* = 0.20) (Supplementary Figure S2). The persistence of very high heterogeneity within the diagnosis-based subgroup (I^2^ = 99.87%), despite minimal heterogeneity among surgery-based studies (I^2^ = 0.14%), suggests that outcome definition alone does not account for the substantial heterogeneity in the overall PM₂.₅ analysis; however, the small number of studies limited the power of this subgroup comparison.

Leave-one-out analyses showed pooled PM₂.₅ estimates ranging from 1.01 to 1.05 and black-carbon estimates ranging from 1.00 to 1.03 (Supplementary Figures S3, S4). For NO₂, pooled estimates remained above unity after sequential omission of individual studies; omission of Chua et al. attenuated the pooled estimate to HR 1.04 (95% CI, 1.00–1.09; *P* = 0.07), whereas omission of Hegelund et al. (8) or Gayraud et al. (10) yielded statistically significant pooled estimates (Supplementary Figure S5).

In additional sensitivity analyses using the Knapp–Hartung adjustment, point estimates were unchanged but confidence intervals were wider. The continuous NO₂ estimate was HR 1.041 (95% CI, 0.982–1.104; *P* = 0.096), and the Q4-versus-Q1 NO₂ estimate was HR 1.094 (95% CI, 0.920–1.302; *P* = 0.096). The overall interpretation of the PM₂.₅ and black-carbon analyses was unchanged.

## 4 Discussion

This systematic review and meta-analysis indicates that the current epidemiological evidence is compatible with an association between chronic air-pollution exposure and cataract-related outcomes, although it remains insufficiently consistent to establish a causal effect of any specific pollutant. Across the broader evidence base, NO₂ showed the most coherent positive pattern, whereas findings for PM₂.₅ varied substantially across populations and outcome definitions, and evidence for black carbon remained limited. The quantitative synthesis reflected this pattern. NO₂ showed the strongest association under conventional random-effects inference, but the estimate was not robust to Knapp–Hartung adjustment, highlighting the uncertainty associated with the small number of contributing studies. Taken together, the available evidence is therefore more appropriately interpreted as indicating a possible pollution-related cataract signal, particularly in relation to traffic- and combustion-related exposures, rather than demonstrating an independent causal effect of NO₂, PM₂.₅, or black carbon. An important source of between-study variation is outcome definition: incident cataract diagnosis and cataract surgery represent related but non-equivalent endpoints, and differences between their effect estimates may reflect differences in the underlying clinical estimand rather than differences in study quality.

This interpretation is broadly consistent with previous evidence syntheses, while the present review provides a more cataract-specific assessment. Grant et al. found limited and heterogeneous evidence regarding cataract within their systematic review of ambient air pollution and age-related eye disease (4). Millen et al. subsequently characterized associations of particulate matter and NO₂ with lens-related outcomes as suggestive, while emphasizing the limited number of longitudinal investigations and the considerable methodological diversity of the available studies (5). A later meta-analysis encompassing several vision disorders reported positive overall associations with PM₂.₅ and NO₂, although the PM₂.₅ association was not statistically significant within the cataract subgroup (6). This distinction matters because pooled estimates across biologically and clinically distinct ocular disorders may capture a broader ocular response to air pollution without necessarily demonstrating an effect on lens opacification itself. By restricting quantitative synthesis to cataract-specific outcomes and sufficiently comparable exposure contrasts, the present review provides a more conservative and clinically focused assessment of this relationship.

The expanding prospective literature has strengthened the evidence base for PM₂.₅, but it has not resolved the inconsistency between studies. In the Canadian Longitudinal Study on Aging, Grant et al. found only a borderline association between PM₂.₅ and self-reported cataract (20). In contrast, Wu et al. reported a positive association between residential PM₂.₅ exposure and incident age-related cataract in UK Biobank, with evidence that associations differed according to cataract phenotype (21). Li et al. subsequently observed increased cataract risk across several long-term PM₂.₅ exposure windows in two Chinese cohorts, accompanied by evidence of an exposure–response relationship (7). A nationwide Danish phenome-wide analysis also identified senile cataract among conditions positively associated with PM₂.₅ (8). However, the prospective 3City-Alienor study did not identify a clear association between PM₂.₅ and cataract surgery (22), and a subsequent analysis of two French nationwide cohorts similarly failed to demonstrate a consistent PM₂.₅ association (10). These apparently conflicting findings make the substantial heterogeneity observed in our PM₂.₅ meta-analysis epidemiologically plausible. Differences in ambient concentration ranges and particle composition, exposure windows, spatial resolution of exposure assessment, population characteristics, confounder adjustment, and cataract ascertainment may all influence the underlying estimand and limit direct comparability between studies.

NO₂ displayed a comparatively more coherent epidemiological pattern, although the present findings should not be interpreted as evidence of an isolated NO₂ effect. Chua et al. reported increasing cataract-surgery risk with higher NO₂ exposure in UK Biobank (23), while the Korean nationwide cohort also identified a positive association between long-term NO₂ exposure and cataract incidence (24). The Danish population-wide study provided additional support for an association with senile cataract (8). In France, Gayraud et al. observed increased cataract-surgery incidence at higher NO₂ concentrations in the 3City-Alienor cohort (22), and their later nationwide analysis found associations predominantly among participants living in urban areas (10). The urban–rural contrast is particularly relevant because NO₂ often represents a broader traffic- and combustion-related exposure environment rather than a toxicologically isolated exposure. Consequently, the observed association may reflect NO₂ itself, correlated co-pollutants, or the combined effects of a traffic-related pollution mixture. Accordingly, the NO₂ findings support an association but do not establish an isolated or pollutant-specific causal effect.

Evidence from studies that could not be incorporated into the principal meta-analyses further supports the need to consider air pollution as a complex exposure rather than as a collection of fully independent pollutants. A recent UK Biobank analysis found stronger associations for a combined air-pollution score than for several individual pollutants, suggesting that joint exposure may contain information not captured by single-pollutant models (9). Another UK Biobank study linked ambient pollution with transitions from a cataract-free state to incident cataract (25). These publications provide complementary evidence but should not be regarded as independent replications because their source populations overlap substantially with other UK Biobank analyses. Findings for ozone illustrate a different form of inconsistency. Earlier Korean data suggested inverse associations between O₃ and cataract (26), whereas a more recent longitudinal analysis of the Chinese Longitudinal Healthy Longevity Survey reported increased cataract risk with chronic O₃ exposure (27). Thus, the earlier suggestion of a potentially protective O₃ association is not supported uniformly by the newer evidence. Studies defining exposure solely by household fuel type constitute a related but methodologically distinct literature and were outside the eligibility criteria of this pollutant-specific review. This source-based evidence has been synthesized separately in a recent systematic review and meta-analysis of unclean cooking fuels and ocular outcomes (28). These findings strengthen the broader hypothesis that chronic combustion-derived exposures may contribute to lens disease, but household smoke and ambient pollution differ substantially in composition, intensity, and exposure pathways and should therefore not be quantitatively treated as equivalent exposures.

Variation in cataract ascertainment is likely to be another important source of heterogeneity. The studies included in the review used outcomes ranging from ophthalmologist-diagnosed lens opacity and cataract subtype grading to self-reported physician diagnosis, registry-defined cataract, and incident cataract surgery. Cataract surgery is a clinically meaningful endpoint because it represents disease severe enough to result in intervention, but it is not equivalent to the biological onset of lens opacification. Surgery occurs downstream of cataract development and depends not only on lens changes and visual impairment but also on patient preferences, clinical thresholds, referral pathways, and access to surgical services (29). Consequently, studies of incident diagnosis and studies of cataract surgery do not necessarily estimate identical biological effects. Accordingly, the pooled analyses should be interpreted as summarizing associations across clinically related cataract outcomes rather than as assuming equivalence between diagnosis and surgery. In the PM₂.₅ subgroup analysis, diagnosis- and surgery-based estimates did not differ statistically (*P* = 0.20), while substantial heterogeneity persisted among diagnosis-based studies themselves. Thus, outcome definition may contribute to the observed heterogeneity but is unlikely to explain it fully. Nevertheless, the small number of contributing studies limits the power of subgroup comparisons and does not exclude genuine outcome-specific differences. Cataract subtype may also be etiologically important. The stronger PM₂.₅ association with non-nuclear cataract reported by Wu et al. differs from the more pronounced nuclear-cataract pattern observed in studies of household combustion exposure (21, 28). This divergence suggests that pollutant source, exposure route, and lens phenotype may interact in ways that are obscured when all cataracts are analyzed as a single outcome.

The epidemiological observations also have biological plausibility, although mechanistic plausibility alone cannot establish causation. Oxidative stress has long been considered a central mechanism in age-related cataractogenesis. Oxidative injury can disrupt the highly reducing environment required to maintain lens crystallins in a soluble state, promote protein modification and aggregation, impair lens epithelial-cell function, and ultimately compromise lens transparency (30). More recent experimental work has provided a mechanism more directly linked to particulate air pollution. Sheng et al. demonstrated structural and metabolic lens injury following long-term PM₂.₅ exposure across experimental models and human lens samples, with evidence implicating disturbed iron homeostasis, glutathione depletion, lipid peroxidation, and ferroptosis-related pathways (3). These findings provide additional biological plausibility for a contribution of particulate pollution to cataractogenesis. They do not, however, resolve the epidemiological heterogeneity observed for PM₂.₅ or establish that associations with NO₂ arise through the same lens-specific mechanisms. The mechanistic literature therefore supports pollution-related oxidative injury as a credible pathway while leaving pollutant-specific causality unresolved.

### 4.1 Strengths and limitations

Several methodological features strengthen the present review. The systematic component considered the full body of eligible evidence rather than limiting interpretation to studies that could be quantitatively pooled. Quantitative synthesis was performed separately according to pollutant and exposure contrast, and continuous and categorical estimates were not combined when they represented materially different estimands. Potentially overlapping source populations were also considered when selecting estimates for pooling, which is particularly important given the growing number of publications derived from UK Biobank and partially overlapping longitudinal cohorts. In addition, random-effects modelling, leave-one-out analyses, and Knapp–Hartung sensitivity analyses allowed the stability of pooled estimates to be evaluated under conditions in which the number of independent studies was small. Collectively, these decisions reduce the risk that apparent precision is generated by inappropriate harmonization or repeated representation of the same underlying population. Important limitations nevertheless remain. All included studies were observational and are therefore susceptible to residual and unmeasured confounding. Because the eligibility criteria were intentionally pollutant-specific, studies defining exposure solely by household fuel type or non-specific cooking smoke were outside the scope of this review; accordingly, the present conclusions should not be generalized to the broader household-air-pollution literature (31, 32). Exposure assessment was also heterogeneous and included land-use regression, monitoring-station measurements, satellite or model-based estimates, regional assignment, and household smoke indicators. Residential exposure estimates cannot completely capture mobility, occupational exposure, indoor sources, or individual time–activity patterns. Outcome heterogeneity further limits comparability, and harmonization of continuous estimates also assumed approximate log-linearity over the rescaled exposure range. Each pollutant-specific meta-analysis contained only a small number of independent studies, limiting statistical precision and precluding meaningful formal evaluation of small-study effects. Finally, repeated publications from large cohorts should not be interpreted as an equivalent number of independent replications, even when they address different exposure formulations or analytical questions.

### 4.2 Future perspectives

Future research should prioritize prospective, phenotype-specific environmental ophthalmic epidemiology rather than repeated analyses of broadly defined “any cataract” outcomes. Repeated residential histories, time-varying or personal exposure assessment, source- and composition-specific particulate metrics, and multipollutant or mixture models could help distinguish individual-pollutant effects from correlated traffic and combustion exposures. Clinically examined nuclear, cortical, and posterior subcapsular cataracts should be analyzed separately whenever feasible, while cataract diagnosis and cataract surgery should remain distinct outcomes. More consistent measurement of ultraviolet exposure, smoking, diabetes, corticosteroid use, socioeconomic conditions, occupational exposures, and other major cataract determinants would reduce residual confounding. Recent advances in portable ophthalmic screening and automated lesion-detection systems in other eye diseases also illustrate the potential of scalable digital phenotyping approaches for future large-scale environmental ophthalmic studies (33, 34). Independent cohorts outside populations that have already generated multiple publications are particularly important. Ultimately, harmonized multicohort analyses, and potentially individual-participant-data approaches, may provide greater etiological resolution for nonlinear exposure–response relationships, pollutant mixtures, susceptible subgroups, and cataract phenotypes.

## 5 Conclusion

The available evidence supports a possible association between chronic air-pollution exposure and cataract-related outcomes, but it does not yet establish a causal effect of any individual pollutant. The most coherent epidemiological signal was observed for NO₂ and related traffic or combustion exposures, although this finding was sensitive to small-study inference and may partly reflect correlated pollutant mixtures. Evidence for PM₂.₅ remained heterogeneous despite positive findings in several large prospective cohorts, while data for black carbon and other pollutants were comparatively limited. Emerging experimental evidence of oxidative and ferroptotic lens injury strengthens the biological plausibility of an association but does not overcome the limitations of the current epidemiological literature. Better exposure characterization, standardized cataract phenotyping, comprehensive confounder control, and replication in independent prospective populations will be necessary to determine whether air pollution contributes meaningfully to cataract development and progression.

## Data Availability

The original contributions presented in the study are included in the article/Supplementary Material, further inquiries can be directed to the corresponding author/s.
