# Supplementary material for: Air pollution exposure and cataract outcomes: a systematic review and pollutant-specific meta-analysis
Source: Front Med (Lausanne). 2026 Oct 1;13:1969759. doi: 10.3389/fmed.2026.1969759 (PMC13668607; doi:10.3389/fmed.2026.1969759)
Supplement: Supplementary file 1 [file Supplementaryfile1.docx]

**Table S1**. Search strategy in databases

| **Database** | **Keywords** | **Results** |
| --- | --- | --- |
| PubMed | ((((((((("Air Pollution"[Mesh]) OR "Particulate Matter"[Mesh]) OR "Nitrogen Dioxide"[Mesh]) OR "Sulfur Dioxide"[Mesh]) OR "Ozone"[Mesh]) OR "Carbon Monoxide"[Mesh]) OR "Soot"[Mesh]) OR (Air Pollutants)) OR (Air Pollutant)) AND ((((("Cataract"[Mesh]) OR (lens opacity)) OR (lens opacities)) OR (cataract surgery)) OR (cataract*)) | 169 |
| Scopus | TITLE-ABS-KEY ( ( "Air Pollution" OR "Particulate Matter" OR "Nitrogen Dioxide" OR "Sulfur Dioxide" OR "Ozone" OR "Carbon Monoxide" OR "Soot" OR "Carbon Black" OR "Black, Carbon" OR "Monoxide, Carbon" OR "Dioxide, Nitrogen" OR "Peroxide, Nitrogen" OR "Particles, Ultrafine" OR "Fiber, Ultrafine" OR "Air Pollutant" OR "Sulfurous Anhydride" OR "Nitrogen Peroxide" OR "Particle, Ultrafine" OR "Air Pollutants" OR "Ultrafine Particle" OR "Ultrafine Fibers" OR "Ultrafine Fiber" OR "Ultrafine Particles" OR "Air Pollutions" OR "Air Quality" OR "Pollution, Air" OR "Air Pollutant" ) AND ( "Cataract" OR "Cataracts" OR "Lens Opacities" OR "Opacities, Lens" OR "Opacity, Lens" OR "Lens Opacity" OR "Pseudoaphakias" OR "Pseudoaphakia" ) ) | 439 |
| Web of Science | TS=(("Air Pollution" OR "Particulate Matter" OR "Nitrogen Dioxide" OR "Sulfur Dioxide" OR "Ozone" OR "Carbon Monoxide" OR "Soot" OR "Carbon Black" OR "Black, Carbon" OR "Monoxide, Carbon" OR "Dioxide, Nitrogen" OR "Peroxide, Nitrogen" OR "Particles, Ultrafine" OR "Fiber, Ultrafine" OR "Air Pollutant" OR "Sulfurous Anhydride" OR "Nitrogen Peroxide" OR "Particle, Ultrafine" OR "Air Pollutants" OR "Ultrafine Particle" OR "Ultrafine Fibers" OR "Ultrafine Fiber" OR "Ultrafine Particles" OR "Air Pollutions" OR "Air Quality" OR "Pollution, Air" OR "Air Pollutant" ) AND ("Cataract" OR "Cataracts" OR "Lens Opacities" OR "Opacities, Lens" OR "Opacity, Lens" OR "Lens Opacity" OR "pseudophakias" OR "pseudoaphakic")) (Topic) | 225 |
| Embase | ('air pollution':ti,ab,kw OR 'particulate matter':ti,ab,kw OR 'nitrogen dioxide':ti,ab,kw OR 'sulfur dioxide':ti,ab,kw OR 'ozone':ti,ab,kw OR 'carbon monoxide':ti,ab,kw OR 'soot':ti,ab,kw OR 'carbon black':ti,ab,kw OR 'black, carbon':ti,ab,kw OR 'monoxide, carbon':ti,ab,kw OR 'dioxide, nitrogen':ti,ab,kw OR 'peroxide, nitrogen':ti,ab,kw OR 'particles, ultrafine':ti,ab,kw OR 'fiber, ultrafine':ti,ab,kw OR 'sulfurous anhydride':ti,ab,kw OR 'nitrogen peroxide':ti,ab,kw OR 'particle, ultrafine':ti,ab,kw OR 'air pollutants':ti,ab,kw OR 'ultrafine particle':ti,ab,kw OR 'ultrafine fibers':ti,ab,kw OR 'ultrafine fiber':ti,ab,kw OR 'ultrafine particles':ti,ab,kw OR 'air pollutions':ti,ab,kw OR 'air quality':ti,ab,kw OR 'pollution, air':ti,ab,kw OR 'air pollutant':ti,ab,kw) AND ('cataract':ti,ab,kw OR 'cataracts':ti,ab,kw OR 'lens opacities':ti,ab,kw OR 'opacities, lens':ti,ab,kw OR 'opacity, lens':ti,ab,kw OR 'lens opacity':ti,ab,kw OR 'pseudoaphakias':ti,ab,kw OR 'pseudoaphakia':ti,ab,kw) | 205 |
| Google scholar | Air Pollution AND Cataract | 28* |

*Results after first evaluation.

Table S2: Risk-of-bias assessment of included studies using ROBINS-E and the WHO air-quality epidemiology risk-of-bias instrument, according to study design.

**A.** ROBINS-E assessment of longitudinal follow-up studies

| **Study** | **D1 Confounding** | **D2 Exposure** | **D3 Selection** | **D4 post-exposure** | **D5 Missing** | **D6 Outcome** | **D7 Reporting** | **Overall** |
| --- | --- | --- | --- | --- | --- | --- | --- | --- |
| Shin et al. (2020) | Some concerns | Some concerns | Low | Low | Low | Low | Some concerns | Some concerns |
| Chua et al. (2021**)** | Some concerns | Some concerns | Some concerns | Low | Low | Low | Some concerns | Some concerns |
| Wu et al. (2022) | Low | Low | Low | Low | Low | Low | Low | Low |
| Li et al. (2023) | Some concerns | Some concerns | Some concerns | Low | Some concerns | Some concerns | Some concerns | Some concerns |
| Hegelund et al. (2024) | Some concerns | Some concerns | Low | Low | Low | Low | Some concerns | Some concerns |
| Gayraud et al. (2025) | Low | Low | Low | Low | Low | Low | Low | Low |
| Li et al. (2025) | Some concerns | Low | Low | Low | Some concerns | Low | Some concerns | Some concerns |
| Wang et al. (2025) | Some concerns | Low | Some concerns | Low | Low | Low | Some concerns | Some concerns |
| Yuan et al. (2025) | Some concerns | Low | Some concerns | Low | Some concerns | Low | Some concerns | Some concerns |
| Zhou et al. (2025) | Some concerns | High | Some concerns | Low | Some concerns | Some concerns | Some concerns | High |
| Gayraud et al. (2026) | Some concerns | Low | Low | Low | Low | Low | Some concerns | Some concerns |

**B.** WHO risk-of-bias assessment of cross-sectional and aggregate studies.

| **Study** | **Confounding** | **Selection bias** | **Exposure assessment** | **Outcome measurement** | **Missing data** | **Selective reporting** | **Overall** |
| --- | --- | --- | --- | --- | --- | --- | --- |
| Choi et al. (2018) | Low | Moderate | Moderate | Low | Moderate | Low | Moderate |
| Grant et al. (2021) | Moderate | Moderate | Moderate | Moderate | Moderate | Low | Moderate |
| Vashist et al. (2020) | Moderate | Moderate | Moderate | Low | Moderate | Low | Moderate |
| Echevarría-Lucas et al. (2024) | High | Low | High | Low | Low | Moderate | High |
| Miao et al. (2025) | High | Moderate | High | Low | Moderate | Moderate | High |

**Figure S1.** Random-effects meta-analysis of high versus low NO₂ exposure using study-specific thresholds. HR for above vs below the study-specific NO₂ threshold

**Figure S2.** Subgroup meta-analysis of the association between PM₂.₅ exposure and cataract according to outcome definition. HR per 1 µg/m³ increase in PM₂.₅

**Figure S3.** Leave-one-out sensitivity analysis of the association between PM₂.₅ exposure and cataract outcomes. HR per 1 µg/m³ increase in PM₂.₅

**Figure S4.** Leave-one-out sensitivity analysis of the association between black carbon exposure and cataract outcomes. HR per study-specific IQR increase in black carbon

**Figure S5.** Leave-one-out sensitivity analysis of the association between NO₂ exposure and cataract outcomes. HR per 10 µg/m³ increase in NO₂
